# Metabolic and molecular evaluation of *Moringa oleifera*-supplemented ketogenic meal replacement in healthy C57BL/6 mice

**DOI:** 10.1038/s41598-025-34443-z

**Published:** 2026-01-28

**Authors:** Ahmed Ibrahim Hassaan, Naglaa M. Ebeed, Amr Fatouh, Hesham Elhariry

**Affiliations:** 1 Department of Food Science, Faculty of Agriculture, Ain Shams University, PO Box 68, Hadayek Shoubra, Cairo, 11241 Egypt; 2 Department of Genetics, Faculty of Agriculture, Ain Shams University, PO Box 68, Hadayek Shoubra, Cairo, 11241 Egypt; 3 Present Address: Food Processing Technology Department, Faculty of Organic Agriculture, Heliopolis University for Sustainable Development,, Cairo, Egypt

**Keywords:** Ketogenic, Diabetes, Moringa, RT-qPCR, Gene expression, C57BL/6 mice, Biochemistry, Diseases, Endocrinology, Physiology

## Abstract

Ketogenic diets, which are high in fat, hold therapeutic promises in obesity and type 2 diabetes and are to be carefully studied in their early stages on healthy mouse models. This study evaluated the physiological, biochemical, histological, and genetic impacts of a *Moringa oleifera*–supplemented ketogenic meal replacement (KMR) compared with a commercial non-ketogenic meal replacement (CMR) and standard chow in female C57BL/6J mice (*n* = 8/group) over 20 weeks. Despite similar caloric intake, KMR-fed mice exhibited ~ 30% lower weight gain than both control and CMR groups, highlighting the role of macronutrient composition over energy content. Insulin sensitivity was preserved across groups, with KMR maintaining fasting glucose, insulin, and HOMA-IR < 0.4. KMR promoted favorable lipid remodeling, including elevated HDL cholesterol (128 ± 6 mg/dL), reduced LDL cholesterol (25 ± 2 mg/dL), and the lowest non-HDL cholesterol, yielding the most favorable HDL: LDL ratio. Liver enzyme analysis revealed hepatoprotective effects in KMR, contrasting with elevated ALT and AST in CMR. Renal biomarkers and the histological observations indicated mild disorder in kidney functions across CMR and KMR groups. At the molecular level, KMR upregulated ketogenesis genes (*Hmgcs2*, *Bdh1*), mitochondrial regulators (*Sirt3*, *Fgf21*), and the anti-inflammatory cytokine *IL10*. Conversely, CMR downregulated *Bdh1*, *Fgf21*, and *IL10* while exerting negligible or nonsignificant effects on *Hmgcs2* and *Sirt3*. Collectively, KMR attenuated weight gain and improved lipid metabolism maintaining insulin and blood glucose levels. This supports its effective dietary management for type 2 diabetes. However, given the observed histological changes, further long-term studies are recommended to confirm the safety of the ketogenic diet on organ tissues.

**Supplementary Information:**

The online version contains supplementary material available at 10.1038/s41598-025-34443-z.

## Introduction

Diabetes mellitus (DM) is a chronic metabolic disorder characterized by sustained hyperglycemia resulting from impaired insulin secretion, insulin resistance, or a combination of both^1^. Diabetes is a global health burden of growing concern, with projections that 783 million people are at risk of developing diabetes by 2045 ^2^. Chronic hyperglycemia contributes to the development of both microvascular (e.g., nephropathy, retinopathy) and macrovascular (e.g., cardiovascular disease) complications via mechanisms involving mitochondrial dysfunction, oxidative stress, and inflammation^3^. While pharmacotherapy remains a cornerstone in diabetes management, there is increasing recognition of nutritional interventions as powerful modulators of metabolic health. Traditional “balanced” meal replacements, commonly prescribed for diabetic patients, often emphasize moderate caloric restriction and controlled carbohydrate intake. However, these formulations may not fully address the underlying metabolic disturbances driving insulin resistance and lipid dysregulation^4^.

The ketogenic diet (KD) has emerged as an alternative dietary approach, characterized by high fat (70–80%), moderate protein (15–20%), and very low carbohydrate intake (< 50 g/day)^5^. This macronutrient profile induces a shift from glycolysis to fat-based metabolism, leading to the hepatic production of ketone bodies, primarily β-hydroxybutyrate (BHB), which serve as alternative energy substrates for peripheral tissues^6^. The metabolic effects of ketosis include reduced insulin demand, enhanced fat oxidation, improved mitochondrial efficiency, and reduced oxidative stress, all of which are beneficial in mitigating diabetic complications^7^.

KD often leads to nutritional deficiencies due to the exclusion of many fruits, grains, and legumes. Therefore, incorporating dietary supplements is essential to maintain optimal health during keto adherence. Key micronutrients such as magnesium, potassium, and certain vitamins (like B-complex and C) are commonly lacking in ketogenic regimens.

Kenig, et al. ^8^ investigated the micronutrient intake and serum concentrations in obese adults adhering to a 12-week KD. They demonstrated that dietary intakes of key micronutrients, including magnesium, calcium, iron, phosphorus, and potassium, consistently fell below the established dietary reference intakes throughout the intervention period. Moreover, they emphasized the importance of strategic food selection and potential supplementation to mitigate the risk of micronutrient deficiencies during prolonged adherence to a KD. Additionally, Storz and Ronco^9^ evaluated nutrient intake among individuals following low-carbohydrate diets. They found that such diets may lead to deficiencies in essential nutrients, including magnesium, vitamin C, and folate. They also recommend that individuals adhering to low-carbohydrate diets consult healthcare professionals and consider supplementation to address potential nutrient gaps. These studies highlighted the necessity of incorporating dietary supplements to maintain optimal health during adherence to a KD. Plant-based supplements, such as *Moringa oleifera*, offer a natural and nutrient-dense solution. *Moringa oleifera* leaf powder is rich in flavonoids and isothiocyanates. It has antioxidant, anti-inflammatory, and insulin-sensitizing properties, which help control blood sugar by boosting insulin signals, lowering oxidative stress, and slowing glucose absorption^10^. Thus, integrating such plant-based supplements can enhance the nutritional adequacy and therapeutic potential of KD.

Additionally, the liver and kidneys play central roles in both diabetes pathogenesis and dietary adaptation. Non-alcoholic fatty liver disease (NAFLD) affects up to 70% of patients with type 2 diabetes^11^, and demonstrates significant improvement under the ketogenic diet intervention. Clinical studies have documented reductions in hepatic fat content alongside normalization of liver enzyme markers, including alanine aminotransferase (ALT) and aspartate aminotransferase (AST), suggesting that KD may offer therapeutic benefits for diabetes-associated hepatic steatosis^12^.

The KD induces a metabolic shift from glycolysis to fatty acid oxidation and ketogenesis, triggering widespread transcriptional reprogramming that impacts energy metabolism, mitochondrial function, and inflammatory responses. This metabolic adaptation is mediated through the upregulation of key genes such as *Bdh1* and *Hmgcs2*, which are central to ketone body synthesis and utilization. *Bdh1* encodes 3-hydroxybutyrate dehydrogenase type 1, the mitochondrial enzyme responsible for the interconversion of β-hydroxybutyrate and acetoacetate, critical for ketone oxidation in extrahepatic tissues^13^. *Hmgcs2* encodes mitochondrial 3-hydroxy-3-methylglutaryl-CoA synthase, the rate-limiting enzyme in hepatic ketogenesis, and is highly responsive to fasting and KD through peroxisome proliferator-activated receptor α (PPARα)-mediated pathways^14–16^.

Mitochondrial function and oxidative metabolism are further supported by genes such as *Sirt3* and *Fgf21*. *Sirt3* encodes a mitochondrial NAD⁺-dependent deacetylase that regulates the activity of metabolic enzymes involved in fatty acid oxidation and reactive oxygen species detoxification. Its expression is upregulated by KD, enhancing mitochondrial efficiency and resilience to metabolic stress^17^. *Fgf21*, a hormone-like growth factor primarily expressed in the liver, acts as a key endocrine regulator of energy homeostasis. KD markedly induces its expression and fasting, acting through the β-Klotho/FGFR1 complex to regulate lipid metabolism, thermogenesis, and insulin sensitivity^18^. In addition to metabolic regulation, KD also influences inflammatory pathways, notably through modulation of *IL10*, which encodes interleukin-10, a potent anti-inflammatory cytokine that counteracts diet-induced inflammatory responses and tissue remodeling. *IL10* treatment has been demonstrated to ameliorate high-fat diet-induced inflammatory processes and associated pathological changes, highlighting its protective role against metabolic inflammation^19^. KD’s ability to enhance endogenous *IL10* production may therefore contribute to its anti-inflammatory profile by preventing diet-induced inflammatory remodeling and maintaining tissue homeostasis in metabolic disorders.

While most studies on diabetic diets use disease-induced models (like streptozotocin-treated or genetically diabetic mice), there’s growing recognition that healthy mice can offer clearer insights when evaluating new dietary interventions, especially before introducing confounding disease variables^20^. These authors noted that healthy C57BL/6J mice fed a high-fat diet can exhibit early signs of glucose intolerance and insulin resistance, making them a valuable model for assessing the nutritional influences that precede the development of type 2 diabetes. In general, early-stage evaluation of dietary interventions in healthy models, before the onset of confounding diabetic pathophysiology, enables a more precise assessment of their metabolic and molecular effects. Accordingly, this study aimed to develop a novel ketogenic meal replacement (KMR) in which *Moringa oleifera* powder is incorporated as part of the overall formulation, and to evaluate its effects on metabolic parameters and the expression of genes related to ketogenesis, mitochondrial function, and inflammation in non-diabetic female C57BL/6 mice. The assessment focused on the KMR as a complete dietary intervention, comparing its efficacy to a standard control diet and a commercial meal replacement (CMR).

## Materials and methods

### Animals and housing

Twenty-four female C57BL/6 mice (6–8 weeks old; 20–22 g) were obtained from the animal house colony of the National Research Centre (NRC), Dokki, Cairo, Egypt. This strain was chosen for its well-characterized genetic background and established susceptibility to diet-induced obesity and diabetes, making it a suitable model for dietary intervention studies. Mice were housed in standard polypropylene cages under control conditions (25 ± 2 °C, 55 ± 5% humidity, 12-h light/dark cycle) with *ad libitum* access to a standard balanced pelleted chow diet and chlorinated tap water. After a 7-day acclimatization period, they were randomly divided into three experimental groups (*n = 8*/group).

### Experimental design

After acclimatization, mice were assigned to one of three dietary regimens for 20 weeks ad libitum. Three diets were used in this experiment (Table 1): a standard control diet, a commercial non-ketogenic meal replacement (CMR), and a formulated ketogenic meal replacement (KMR). Diets were freshly prepared daily and provided at 10:00 am in sterilized dishes. Food intake was recorded by weighing uneaten food after 24 h, and body weight was monitored weekly.

### Diet composition

**Fig. 1:**
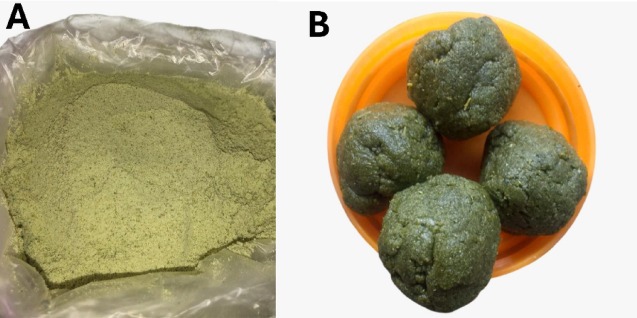
*Moringa oleifera*-supplemented ketogenic meal replacement powder (**A**) and dough (**B**).

The control diet composition was adopted from Kamel et al.^21^. The CMR is a commercial, ready-to-use meal from which dough is prepared according to the preparation instructions on the commercial package. The KMR was formulated according to the macronutrient profile described by Crosby et al.^22^. The macronutrient composition and energy content of all diets are specified in Table 1. Five grams of dried *Moringa oleifera* leaf powder were incorporated into the base ingredients of the ketogenic meal and thoroughly blended to achieve the final macronutrient composition outlined in Table 1. The daily caloric intake for each diet was calculated based on caloric density using Atwater conversion factors (4 kcal/g for carbohydrates and proteins, 9 kcal/g for fats) to ensure accurate energy assessment across groups. Daily intake was standardized to ~ 15 kcal/mouse/day, consistent with C57BL/6 energy requirements. A portion of each diet was ​​mixed with 20% water to form a dough that was fed to the mice (Fig. 1).

**Table 1:** Macronutrient composition and energy content of experimental diets.

Component	Control		CMR		KMR	
	g/kg diet	kcal%	g/kg diet	kcal%	g/kg diet	kcal%
**Protein**	220 (Milk protein concentrate)	23%	137 (Milk protein concentrate & Soy protein isolate)^$^	20%	250 (Whey protein isolate)	20%
**Carbohydrates**	631 (Corn starch)	67%	494 (Hydrolysate corn starch & Maltodextrins)^$^	73%	125 (Glucose syrup - Mono- and disaccharides)	10%
**Fat**	43 (Corn Oil)	10%	22 (High oleic sunflower, soy, and canola oils)^$^	7%	391 (Refined palm oil)	70%
**Vitamin mix**	10		As described on the commercial package		5 Moringa leaf powder^*^	
**Mineral mix**	40					
**Metabolizable energy (kcal/g diet)**	3.8		4.0		5.0	

CMR: Commercial Meal Replacement, KMR: Ketogenic Meal Replacement. Caloric percentages were calculated according to Atwater conversion factors (protein: 4 kcal/g; carbohydrate: 4 kcal/g; fat: 9 kcal/g) relative to the total caloric intake. * Moringa leaf powder was added to KMR as a natural source of vitamins and minerals, comprising approximately 0.5% of the total formulation. ^$^ Mixtures within the components of the commercial meal according to the label of the package.

### Ethical statement

All animal procedures were approved by the Ethics Committee of the National Research Centre (NRC), Institutional Animal Care, Cairo, Egypt . All experiments were performed in accordance with the National Institutes of Health Guide for the Care and Use of Laboratory Animals (NIH Publications No. 8023, revised 1978), the Egyptian guidelines for the care and use of laboratory animals, and the ARRIVE guidelines^23^. Anesthesia and euthanasia procedures complied with the American Veterinary Medical Association (AVMA) Guidelines for the Euthanasia of Animals^24^. Every effort was made to minimize animal suffering during the experimental period.

### Blood and tissue sampling

Overnight fasted mice were weighed at the end of the treatment period, and blood samples were obtained from the tail tip to measure the fasting blood glucose (FBG). Mice were then anesthetized via intraperitoneal injection of ketamine (120 mg/kg) and xylazine (16 mg/kg)^25,26^. Blood samples were collected via cardiac puncture, incubated on ice for 30–60 min, and centrifuged at 3000 ×g for 15 min at 4 °C. After that, serum was collected. Serum aliquots were stored at − 80 °C for biochemical analysis.

Following blood collection, mice were euthanized by cervical dislocation in compliance with institutional guidelines. Liver and kidneys were excised, rinsed in cold saline, blotted, weighed, and divided for analysis: one portion fixed in 10% neutral buffered formalin for histopathology, and the remainder snap-frozen in liquid nitrogen for Molecular assay. Frozen tissues were homogenized in ice-cold PBS (pH 7.4) and stored at − 80 °C until analysis. Tissues were collected into 10% histology-grade formalin or snap frozen in liquid nitrogen and stored at − 80 °C.

## Biochemical assessment

### Glucose homeostasis parameters

The concentration of fasting serum glucose (FBG) was determined using a commercial kit (Bio Med Diagnostic INC, USA). Serum insulin levels were measured using a rat-specific enzyme-linked immunosorbent assay (ELISA) kit (EMD Millipore, USA), following the manufacturer’s instructions. Homeostasis Model Assessment-Insulin Resistance (HOMA-IR) was calculated by the equation of Caumo, et al. ^27^:

HOMA − IR = [Fasting Insulin (µIU/mL) ×Fasting Glucose (mg/dL)] / (22.5 × 18).

### Lipid profile and liver enzymes

To evaluate liver function and lipid metabolism, the serum levels of several key parameters were measured. A standard enzymatic colorimetric method was used to determine the serum activity of alanine aminotransferase (ALT) and aspartate aminotransferase (AST), as well as the serum concentrations of triglycerides (TG), total cholesterol (TC), high-density lipoprotein cholesterol (HDL-C), and low-density lipoprotein cholesterol (LDL-C). The measurements were performed using a commercial kit (Bio Med Diagnostic INC, USA) following the manufacturer’s instructions. Additionally, very low-density lipoprotein cholesterol (VLDL-C) and non-HDL cholesterol (Non-HDL-C) were calculated. The HDL risk factor was also determined. All measurements were conducted following established laboratory protocols (Nagahama Life Science Laboratory, Shiga, Japan). HDL Risk Factor was calculated as the total cholesterol/HDL ratio.

### Kidney function

Serum creatinine and blood urea nitrogen (BUN) levels were determined using a colorimetric endpoint method, according to the manufacturer’s instructions (Diamond Diagnostics, MDSS GmbH, Hannover, Germany).

### Histopathological examination

The fixed specimens of the liver and Kidney were processed overnight for dehydration, clearing, and impregnation using an automatic tissue processor (Sakura, Japan). The specimens were embedded in paraffin blocks using an embedding station (Sakura, Japan), and serial sections of 4 μm thickness were cut using a microtome (Model RM2245, Leica Biosystems, Wetzlar, Germany). We used an autostainer (Model 5020, Leica Biosystems, Wetzlar, Germany) for Hematoxylin & Eosin staining of the sections. The mounted specimens were observed and scored under light microscopy. For a semi-quantitative comparison of the structural changes, the abnormalities in the tissue sections were graded from 0 (normal structure) to 3 (severe pathological changes).

### Real-Time PCR of ketogenesis genes

Reverse transcription quantitative PCR (RT-qPCR) was performed as previously described^28,29^. The RNeasy Mini Kit (Qiagen^®^, Germany) was used to isolate total RNA from the liver according to the manufacturer’s instructions, and the nanodrop was used to assess the extracted RNA’s integrity and concentration. The miScript II RT Kit was used to do reverse transcription following the manufacturer’s instructions. The tissue expressions of *Bdh1*, *Hmgcs2*,* IL10*,* Sirt3*, and *Fgf21* were quantified in the cDNA relative to the reference gene β-actin using Rotor Gene SYBR Green PCR Kit (Qiagen^®^, USA).

The conditions for quantitative PCR amplification were set up as follows: a 10-minute initial denaturation at 95 °C was followed by 45 PCR cycles of denaturation at 95 °C for 20 s, annealing at 59 °C for 20 s, and extension at 70 °C for 15 s. For normalization, the housekeeping gene Beta-actin was used as a reference gene. The primers used in the present study are presented in Table 2. Using the 2-ΔΔCt method, the relative change in mRNA expression in the samples was determined^30^.

**Table 2:** Primer sets for gene expression.

Gene name	Primer sequence	Ref seq number	Amplicon size, bp	Annealing temperature
***Bdh1***	F: GTTAACAACGCAGGCATCTC R: AACTTGGTGATGCAGTATGG	NM_00112268 3.1	215pb	59 °C
***Fgf21***	F: GGTACCTCTACACAGATGAC R: AAGTGAGGCGATCCATAGAG	NM_020013	208pb	59 °C
***Hmgcs2***	F: GCTGCCAACTGGATGGAG R: GTCGTACGCGTTCTCCATGT	NM_008256	195pb	59 °C
***Sirt3***	F: CCGACATTGTGTTCTTTGG R: TCAAGCTGGCAAAAGGCTC	NM_00117780 4	126pb	59 °C
***Il10***	F: CAGGGCCCTTTGCTATGGTG R: CGGCTGGGGGATGACAGTAG	NM_010548	168pb	59 °C
***Actb***	F: AGCCATGTACGTAGCCATCC R: TCCCTCTCAGCTGTGCTGGTGAA	NM_007393	231pb	59 °C

*Bdh1*: 3-hydroxybutyrate dehydrogenase, type 1, *Fgf21*: Fibroblast growth factor 21, *Hmgcs2*: 3-hydroxy-3- methylglutaryl-Coenzyme A synthase 2, *Sirt3*: Sirtuin 3, *Il10*: Interleukin 10, *Actb*: Beta actin.

### Statistical analyses

The data were presented as mean ± standard deviation (SD) and subjected to one-way analysis of variance (ANOVA) for group comparisons. At *p* < 0.05, the p-value was considered significant. Statistical analyses were done using GraphPad Prism (version 7, GraphPad Software INC., La Jolla, CA, United States).

## Results and discussion

### Body weight gain

Body weight progression and net weight gain were monitored across control, commercial meal replacement (CMR), and ketogenic meal replacement (KMR) groups over a 20-week feeding period (Table 3). At baseline, no significant differences (*p* > 0.05) in body weight were observed among the groups, confirming successful randomization and excluding initial body weight as a confounding factor. After 20 weeks, all groups exhibited a significant increase in body weight compared with baseline (*p* < 0.001). The highest weight was recorded in the CMR group (26.7 ± 1.0 g). The control and CMR groups showed nearly identical relative weight gains (23.2 ± 2.2% and 23.0 ± 2.3%, respectively), indicating that the CMR diet did not alter weight progression compared with the standard chow diet. In contrast, the KMR group displayed a significantly attenuated weight gain of 16.5 ± 2.0%, corresponding to an approximate 30% reduction compared with both the control and CMR groups, despite similar caloric intake (*p* < 0.001). The KMR used in the present study, fortified with *Moringa oleifera*, effectively reduces weight gain. This may occur through enhanced fat oxidation, suppressed lipogenesis, and improved metabolic efficiency via ketosis^31–33^. This highlights the critical role of macronutrient composition over product form in achieving metabolic advantages^34^. The integration of ketogenic nutrition with functional botanicals offers a promising strategy for the prevention of obesity. In general, the contrast between CMR and KMR responses is indicative of the pivotal role played by macronutrient source and ratio in modulating weight dynamics and metabolic health. The reduced feeding efficiency observed in the KMR group is interpreted as supporting the therapeutic potential of ketogenic dietary strategies for obesity prevention and management through metabolic reprogramming^31^.

**Table 3:** Body weight progression and net weight gain in mice across dietary groups over a 20-week feeding period.

Groups	Initial Weight (g)	Final Weight (g)	Weight gain (%)
Control	19.8^a^ ± 1.7	24.4^a^ ± 0.9	23.2^a^ ± 2.2%
CMR	21.7^a^ ± 2.0	26.7^a^ ± 1.0	23.0^a^ ± 2.3%
KMR	21.8^a^ ± 1.6	25.4^a^ ± 2.5	16.5^a^ ± 2.0%

Mice were fed on a basal diet (Control), Commercial Meal Replacement (CMR), and Ketogenic Meal Replacement (KMR). Data were expressed as Means ± SD (*n = 8*). Values with the same superscript letter within each column are not significantly different (*P > 0.05*). Statistical analysis was performed using one-way ANOVA followed by Tukey post-hoc test.

### Organs weight

The absolute and relative weights of the liver, kidney, and spleen showed no statistically significant differences (*p > 0.05*) among the control, CMR, and KMR groups (Table 4). All relative values remained within the normal physiological range for healthy female C57BL/6 mice, indicating the absence of organ enlargement (hypertrophy) or shrinkage (atrophy) due to dietary intervention. This minimal variation suggests that the feeding regimens had little to no effect on organ mass when adjusted for body weight. These findings are consistent with previous reports that moderate dietary modifications, including ketogenic and meal replacement approaches, do not typically cause substantial changes in absolute or relative organ weights in healthy rodents^33,35^. Overall, both CMR and KMR diets preserved organ structural integrity, reinforcing their safety profile in long-term feeding studies.

**Table 4:** Absolute and relative organ weight of mice after feeding on different diets for 20 weeks.

Group	Organ weight					
	Liver		Kidney		Spleen	
	Absolute (g)	Relative (%)	Absolute (g)	Relative (%)	Absolute (g)	Relative (%)
**Control**	1.25^a^ ± 0.09	5.12^a^ ± 0.37	0.27^a^ ± 0.02	1.10^a^ ± 0.06	0.10^a^ ± 0.01	0.41^a^ ± 0.03
**CMR**	1.31^a^ ± 0.12	4.91^a^ ± 0.42	0.29^a^ ± 0.02	1.08^a^ ± 0.07	0.11^a^ ± 0.01	0.40^a^ ± 0.04
**KMR**	1.18^a^ ± 0.11	4.65^a^ ± 0.38	0.26^a^ ± 0.03	1.02^a^ ± 0.05	0.09^a^ ± 0.01	0.36^a^ ± 0.02

Mice were fed on a basal diet (Control), Commercial Meal Replacement (CMR), and Ketogenic Meal Replacement (KMR). Data were expressed as Means ± SD ( *n = 8)*. Values with the same superscript letter within each column are not significantly different (*P > 0.05*).

### Glucose homeostasis parameters

Fasting blood glucose (FBG), fasting plasma insulin, and HOMA-IR are commonly used to assess baseline insulin sensitivity and glucose homeostasis in rodent studies, including mice^36^. Results of the current study demonstrate that all experimental groups maintained FBG levels within the normal physiological range for healthy C57BL/6J female mice (Table 5). The control group recorded 92.1 ± 4.2 mg/dL, while the CMR group showed a modest reduction to 81.9 ± 3.8 mg/dL, and the KMR group demonstrated a slight elevation to 97.3 ± 5.1 mg/dL. Although statistical differences were observed between treatment groups, all values remained within acceptable normal limits for healthy mice (70–110 mg/dL), indicating that both meal replacement formulations effectively maintained adequate glycemic control without inducing hyperglycemia or hypoglycemia^37^.

Insulin levels remained consistently within normal physiological ranges across all experimental groups (0.40 ± 0.0 µIU/mL), with no significant differences detected among control, CMR, and KMR groups (Table 5). This stability suggests that neither meal replacement formulation adversely affects pancreatic cell function or insulin secretion capacity in healthy mice. Murata, et al. ^38^ examined C57BL/6 mice fed a ketogenic diet (KD) for 6 or 14 days and observed that blood insulin levels were not decreased in mice fed KD for 14 days.

The homeostasis model assessment index for insulin resistance (HOMA-IR) remained consistently low (< 0.4) across all treatment groups, confirming preserved insulin sensitivity and normal glucose homeostasis regardless of the meal replacement formulation administered^36^. These findings indicate that both CMR and KMR formulations are metabolically safe and do not compromise insulin function or glucose tolerance in healthy subjects.

A ketogenic diet, which severely restricts carbohydrate intake, prompts the body to maintain glucose levels through gluconeogenesis, producing glucose from amino acids and glycerol. In the KMR group, a slight increase in fasting blood glucose (FBG) likely reflects this adaptive process, which is a normal response to carbohydrate restriction. Since FBG remained within the normal range and insulin sensitivity (measured by HOMA-IR) was preserved, this does not indicate a negative outcome. Instead, it demonstrates metabolic flexibility, where the body effectively regulates glucose through alternative pathways, a hallmark of healthy adaptation to a ketogenic diet^39^.

**Table 5:** Changes in glucose homeostasis parameters in C57BL/6J female mice fed different diets.

Groups	FBG (mg/dL)	Insulin (µIU/mL)	HOMA–IR
Control	92.1^a^ ± 4.2	0.40 ± 0.0	< 0.4 ± 0.0
CMR	81.9^b^ ± 3.8	0.40 ± 0.0	< 0.4 ± 0.0
KMR	97.3^a^ ± 5.1	0.40 ± 0.0	< 0.4 ± 0.0

Mice were fed on a basal diet (Control), Commercial Meal Replacement (CMR), and Ketogenic Meal Replacement (KMR). Data are expressed as mean ± SD (*n = 8*). Statistical comparisons were performed using one-way ANOVA followed by Tukey’s post-hoc test (*p* < 0.05). Means with the same superscript small letter in the same column are not significantly different. Fasting blood glucose (FBG) concentrations were measured from the tail vein using a handheld glucometer after 12-hour overnight fasting.

### Lipid profile analysis

Lipid profile evaluation across the experimental groups (Control, CMR, and KMR) revealed distinct and clinically relevant metabolic patterns (Table 6). There were no significant differences in total cholesterol between the control and CMR groups, which were 105 ± 5 and 104 ± 4, respectively. On the other hand, the KMR group exhibited the most pronounced cardioprotective changes, with total cholesterol levels of 167 ± 8 mg/dL (*p < 0.05*), driven primarily by a remarkable increase in high-density lipoprotein (HDL) cholesterol (128 ± 6 mg/dL) compared to the Control (28 ± 3 mg/dL) and CMR (56 ± 4 mg/dL) groups. Notably, the combination of high HDL and low LDL in the KMR group resulted in the lowest non-HDL cholesterol levels (39 ± 2 mg/dL; *p* < 0.05 vs. control: 77 ± 4 mg/dL), representing the lowest calculated cardiovascular risk burden among all groups. An improvement in the HDL: LDL ratio was recorded in the KMR group (5.12:1) compared to the Control group (0.47:1), indicating enhanced reverse cholesterol transport and reduced atherogenic potential.

**Fig. 2:**
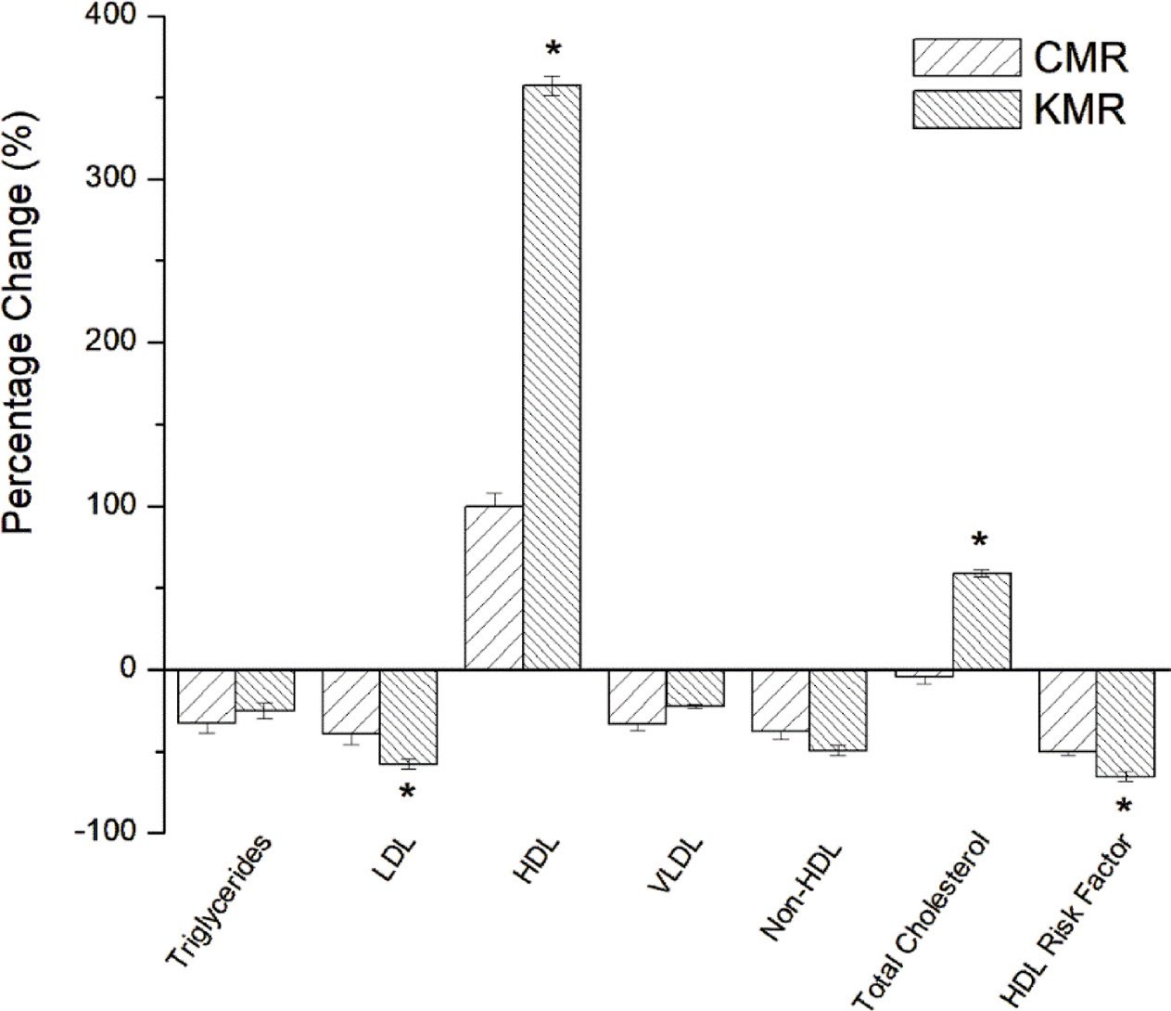
Percentage change in lipid profile parameters in C57BL/6J female mice after consuming Commercial Meal Replacement (CMR) and Ketogenic Meal Replacement (KMR). Statistical significance was determined using one-way ANOVA followed by Tukey’s post-hoc test (*p < 0.05*). Columns with an asterisk (*) indicate a significant difference within the same parameter.

As shown in Fig. 2, the substantial HDL elevation in the KMR group was accompanied by a significant 58% reduction in low-density lipoprotein (LDL) cholesterol (25 ± 2 mg/dL in KMR vs. 59 ± 4 mg/dL in Control). While the CMR group achieved the greatest triglyceride reduction (33%) compared to the control, the KMR group also demonstrated a moderate reduction (25%). By calculating the percentage change in HDL cholesterol, it is clear that the percentage increase in HDL cholesterol was 357%, while the percentage decrease in LDL cholesterol was 58%, as the main distinguishing features of the mice fed the ketogenic diet prepared in this study (Fig. 2).

Previous literatures stated that the ketogenic diets alone often lower triglycerides but may increase LDL and total cholesterol depending on diet composition and intervention duration^40,41^. Moreover, Sharman et al.^42^ stated that the HDL cholesterol tended to increase (+ 11.5%) with the short-term ketogenic diet (6 weeks).

**Table 6:** Lipid profile in C57BL/6J female mice fed different diets.

Parameter^†^ (mg/dL)	Control	CMR	KMR
**Triglycerides**	92 ^a^ ± 6	62 ^b^ ± 5	69 ^b^ ± 4
**LDL**	59 ^a^ ± 4	36 ^b^ ± 3	25 ^c^ ± 2
**HDL**	28 ^c^ ± 3	56 ^b^ ± 4	128 ^a^ ± 6
**VLDL**	18 ^a^ ± 1	12 ^c^ ± 1	14 ^b^ ± 1
**Non-HDL**	77 ^a^ ± 4	48 ^b^ ± 3	39 ^c^ ± 2
**Total Cholesterol**	105 ^b^ ± 5	104 ^b^ ± 4	167 ^a^ ± 8
HDL Risk Factor*	3.75 ^a^ ± 0.3	1.86 ^b^ ± 0.2	1.30 ^c^ ± 0.1

Data are expressed as mean ± SD (*n = 8* per group), CMR: Commercial Meal Replacement; KMR: Ketogenic Meal Replacement. Statistical significance was determined using one-way ANOVA followed by Tukey’s post-hoc test (*p < 0.05*). Means with the same superscript small letter in the same row are not significantly different. * HDL Risk Factor refers to a calculated ratio of total cholesterol/HDL. † Lipid fractions were calculated as follows: Total cholesterol = HDL + LDL + VLDL; Non-HDL cholesterol = LDL + VLDL (or Total cholesterol − HDL).”

### Liver enzymes

**Fig. 3:**
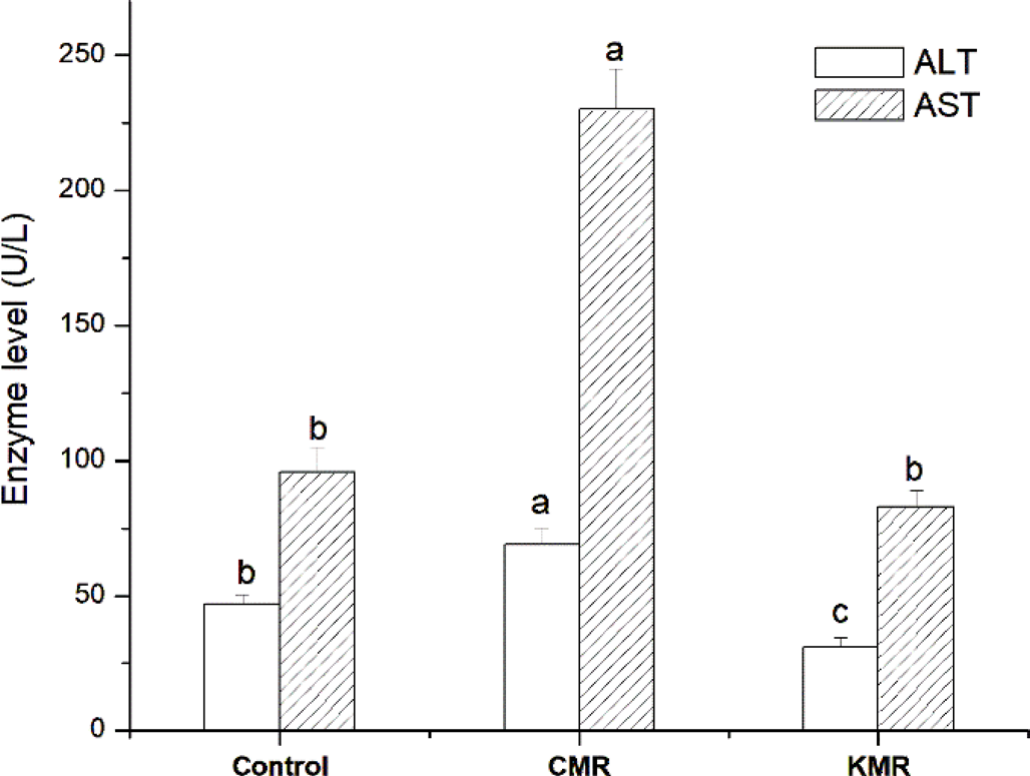
Changes in liver enzyme levels (ALT and AST) in C57BL/6J female mice after consuming Commercial Meal Replacement (CMR) and Ketogenic Meal Replacement (KMR). Statistical significance was determined using one-way ANOVA followed by Tukey’s post-hoc test (*p < 0.05*). Means with the same superscript small letter for the same enzyme are not significantly different.

Alanine aminotransferase activity (ALT) and aspartate aminotransferase activity (AST) were determined in the blood serum of the tested groups. In healthy mice, the normal physiological ranges for ALT are approximately 17–70 U/L and for AST are about 40–120 U/L. In the control group, ALT (47 U/L) and AST (96 U/L) levels remained within these ranges, confirming a healthy baseline (Fig. 3). Similarly, the KMR group showed ALT (31 U/L) and AST (83 U/L) values well within normal limits, supporting the proposed protective or stabilizing effect on liver function. In contrast, the CMR group exhibited ALT levels (69 U/L) at the upper limit of normal, while AST levels (230 U/L) were markedly elevated, more than double the normal range, suggesting substantial hepatocellular stress or damage. The statistically significant reductions in both ALT and AST levels in the KMR group compared to the CMR group indicate a protective or stabilizing effect on liver enzymes under the KMR intervention. This might be attributed to the presence of phenols and flavonoids in the KMR formulation.

### Serum creatinine and blood Urea nitrogen

**Fig. 4:**
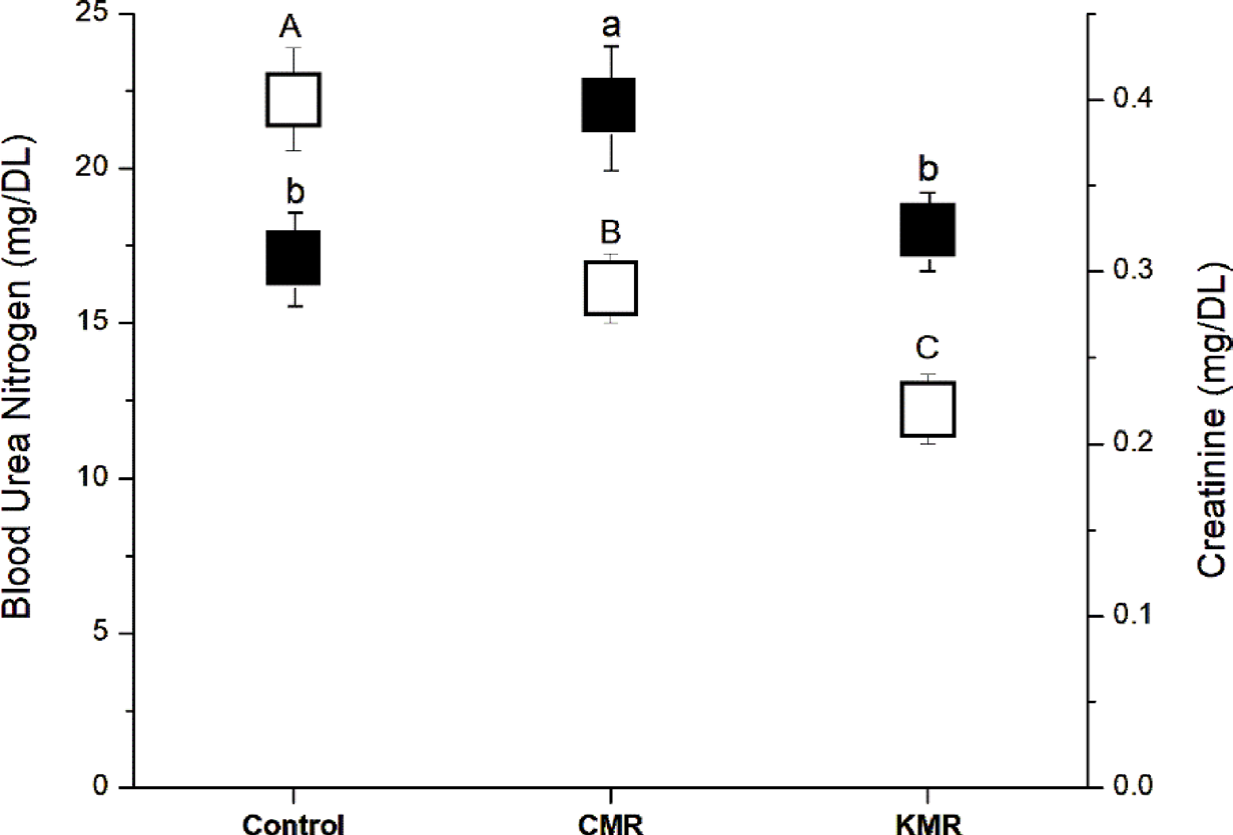
Changes in serum creatinine (open box) and blood urea nitrogen (dark box) levels in C57BL/6J female mice after consuming Commercial Meal Replacement (CMR) and Ketogenic Meal Replacement (KMR) for 20 weeks. Statistical significance was determined using one-way ANOVA followed by Tukey’s post-hoc test (*p < 0.05*). Means with the same superscript small letter for the same parameter are not significantly different.

Both the CMR and KMR groups showed significantly lower creatinine levels compared with the control group, with insignificant differences between the CMR and KMR groups (Fig. 4, open bar). Serum creatinine serves as a primary biomarker for renal function assessment, and the observed reductions in both intervention groups suggest improved kidney function^43,44^. Previous studies have demonstrated that ketogenic diets are well-tolerated with no gross changes to serum creatinine levels, supporting the safety profile of ketogenic dietary interventions^45^. Research indicates that low-carbohydrate diets, including very low-calorie ketogenic diets, can be safely implemented without deleterious renal effects^46,47^.

Regarding blood urea nitrogen (BUN) levels, the CMR group demonstrated significantly higher BUN levels compared to both the control and KMR groups (Fig. 4, dark bar). Only the control and KMR groups showed similar BUN levels with insignificant differences between them. Blood urea nitrogen represents the primary metabolite derived from dietary protein and tissue protein turnover, providing complementary information to creatinine for comprehensive renal function evaluation^43^. The elevated BUN levels in the CMR group may reflect altered nitrogen handling due to caloric restriction with high carbohydrate load (hydrolyzed corn starch and maltodextrins), potentially inducing relative dehydration and prerenal azotemia. Concurrent hepatic stress (elevated ALT and AST) may further contribute via disrupted urea cycle dynamics. In contrast, the ketogenic diet supplemented with Moringa oleifera maintained BUN levels comparable to controls, likely due to its nephroprotective antioxidant effects.

Previous research has demonstrated that *Moringa oleifera* significantly ameliorates increases in serum BUN and creatinine levels, which may explain the favorable renal function parameters observed in the KMR group^48^. Studies have shown that *Moringa oleifera* exhibits nephroprotective effects and effectively attenuates deleterious renal effects via alleviation of tissue oxidative stress^48,49^. The maintained BUN levels in the KMR group, comparable to controls, suggest that *Moringa oleifera* supplementation may provide renal protection during ketogenic dietary intervention through its antioxidant and anti-inflammatory properties^50,51^.

### Histopathological assessment of hepatic tissue

**Fig. 5:**
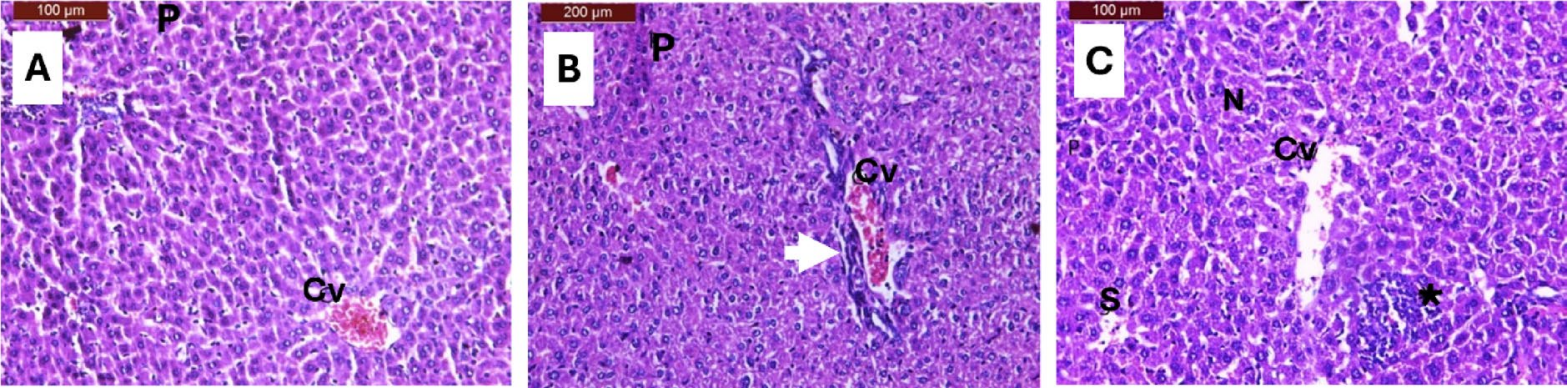
Liver sections in C57BL/6J female mice. (**A**) The control group showed nearly normal liver structure, with slight congestion of the central vein (Cv). (**B**) CMR group showed moderate histological changes, congestion of the central vein (Cv) with inflammatory cells around the central vein (white arrow), and a few pyknotic nuclei (P). (**C**) KMR group showed moderate histological changes in the liver structure of hepatocytes, slight dilatation of the central vein (Cv) and blood sinusoids (S), focal inflammatory cells (star), and normal nuclei (N). Sections were stained with haematoxylin and eosin (H & E).

The histopathological examination of liver tissue from female C57BL/6J mice revealed distinct morphological patterns across experimental groups, providing critical insights into the hepatoprotective potential of ketogenic dietary interventions supplemented with *Moringa oleifera* (Fig. 5). Control animals demonstrated preserved hepatic architecture with minimal pathological alterations, characterized by slight central vein congestion and occasional inflammatory cell infiltrates, consistent with normal physiological variations observed in laboratory mice^52,53^.

The ketogenic diet with *Moringa oleifera* supplementation (KMR) group exhibited moderate structural modifications while maintaining hepatocellular integrity. Histological analysis revealed mild dilation of central veins and hepatic sinusoids, accompanied by focal inflammatory cell clusters and predominantly normal nuclear morphology (Fig. 5). These findings align with previous studies demonstrating the hepatoprotective properties of *Moringa oleifera*, which contains bioactive compounds including quercetin, chlorogenic acid, and β-sitosterol that contribute to anti-inflammatory and antioxidant effects^51,54^. The preservation of normal nuclear architecture in the KMR group suggests minimal hepatocellular stress, supporting the protective role of moringa supplementation in ketogenic dietary protocols.

In contrast, the conventional calorie-restricted (CMR) group demonstrated more pronounced hepatic alterations, including central vein congestion with extensive pericentral inflammatory infiltration and the presence of pyknotic nuclei indicative of cellular damage (Fig. 5). These morphological changes corresponded with significantly elevated hepatic transaminase levels observed in the CMR group compared to substantially lower enzyme activities in the KMR group, confirming hepatocellular damage in the calorie-restricted intervention^55^. The correlation between histopathological findings and biochemical markers reinforces the clinical significance of these observations and demonstrates the superior hepatic preservation achieved through ketogenic dietary intervention with *Moringa oleifera* supplementation.

Histopathological evidence demonstrates that ketogenic dietary intervention with *Moringa oleifera* supplementation offers superior hepatoprotective effects compared to conventional calorie restriction. The maintained hepatocellular integrity reduced inflammatory infiltration, and preservation of normal nuclear morphology in the KMR group, coupled with favorable biochemical profiles observed in the present study, support the therapeutic potential of this dietary approach for optimizing metabolic health while minimizing hepatic complications.

### Histopathological assessment of renal tissue

**Fig. 6:**
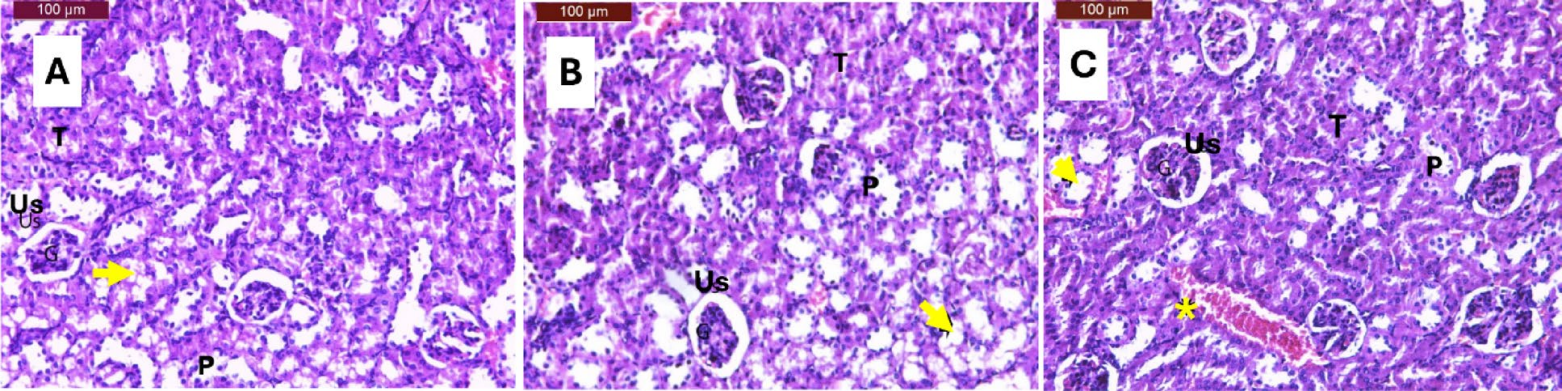
Kidney sections in C57BL/6J female mice. (**A**) The control group showed nearly normal kidney structure, with mild dilatation of the urinary space (Us). (**B**) CMR group showed mild histological changes, atrophic glomeruli, and mild dilatation of the urinary space (Us). (**C**) KMR group showed mild histological changes, normal tubules (T), others showed degeneration (arrowhead), pyknotic nuclei (P), and interstitial haemorrhage (star). Sections were stained with haematoxylin and eosin (H & E).

Histopathological evaluation of kidney sections from female C57BL/6J mice revealed distinct morphological patterns across experimental groups, providing insights into the differential renal effects of dietary interventions (Fig. 6). The control group demonstrated baseline renal changes characteristic of the experimental model, including mild glomerular atrophy, slight dilation of urinary space, and mixed tubular morphology with some regions showing normal architecture while others exhibited degenerative changes and pyknotic nuclei. These findings establish the baseline renal status against which interventions can be evaluated^43,46^.

The conventional calorie-restricted (CMR) group exhibited histological changes like the control group, with mild glomerular atrophy, slight urinary space dilation, mixed tubular architecture showing both normal and degenerative patterns, and the presence of pyknotic nuclei. Despite the elevated BUN levels observed biochemically in the CMR group compared to controls and KMR groups, the histopathological changes remained relatively mild and comparable to controlling animals. This suggests that while the CMR intervention may have affected nitrogen metabolism, as evidenced -by biochemical parameters, the structural renal damage remained limited during the experimental period^44,45^.

In contrast, the ketogenic diet with *Moringa oleifera* supplementation (KMR) group presented more pronounced histological alterations, including moderate changes characterized by glomerular atrophy, urinary space dilation, mixed tubular morphology, and notably, the presence of interstitial hemorrhage alongside pyknotic nuclei (Fig. 6). Previous studies have reported that high-dose *Moringa oleifera* supplementation can result in mild hemorrhage in interstitial spaces, which aligns with the current histopathological findings^56^. The presence of interstitial hemorrhage in the KMR group represents a concerning finding that was absent in both the control and CMR groups.

#### Real-Time PCR of ketogenesis genes

**Fig. 7:**
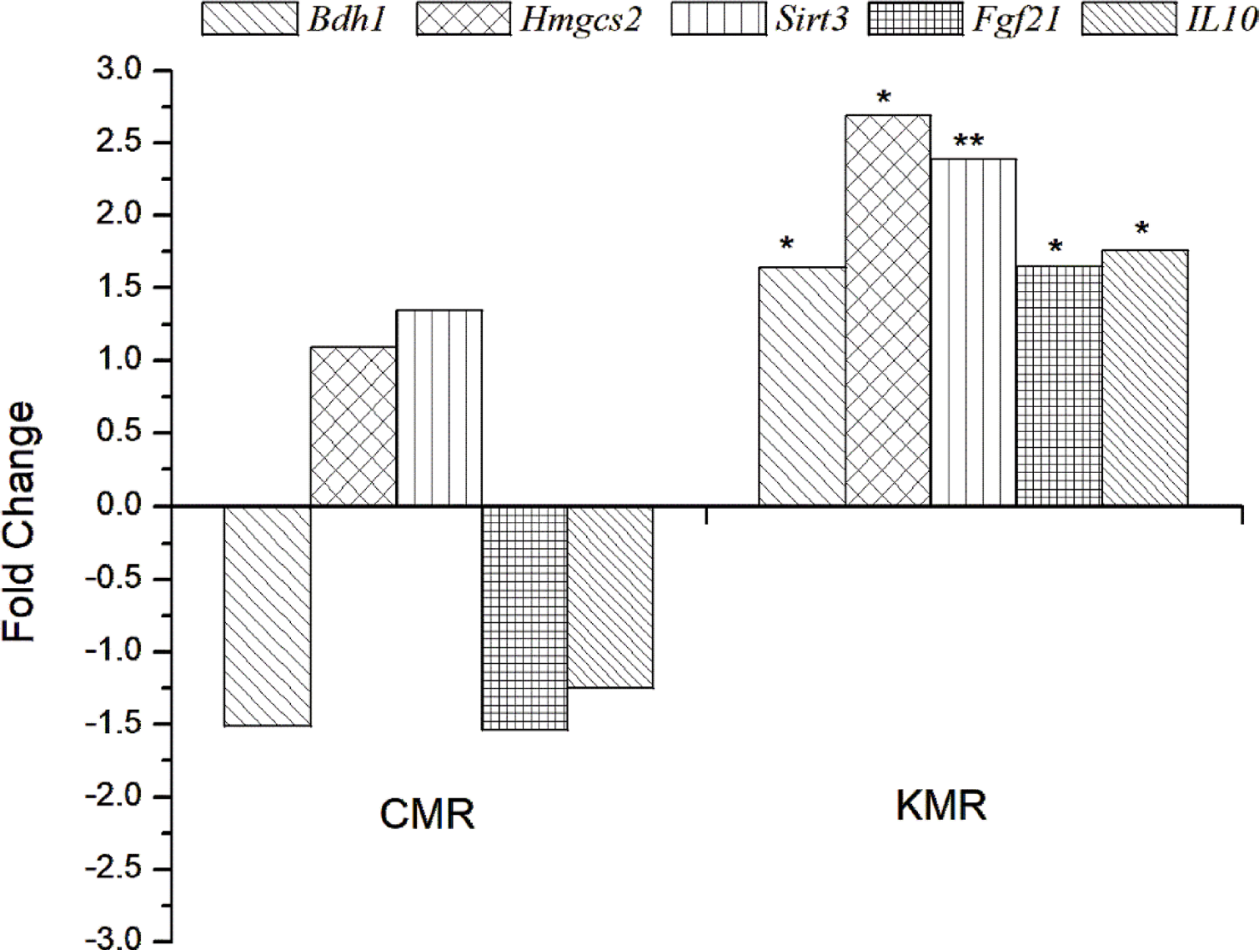
Fold change expressions of *Bdh1*,* Hmgcs2*,* Sirt3*,* Fgf21*,* and IL10* mRNAs using RT-qPCR from liver in bar diagram normalized with Beta actin internal control in CMR and KMR group.in C57BL/6J female mice after consuming Commercial Meal Replacement (CMR) and Ketogenic Meal Replacement (KMR). *p < 0.05, **p < 0.01.

Gene expression analysis of five key regulators of ketogenesis and metabolic adaptation (*Bdh1*,* Hmgcs2*,* Sirt3*,* Fgf21*,* and IL10*) revealed distinct transcriptional responses among the experimental groups (Control, CMR, and KMR). Real-Time PCR raw data is also available as supplementary data (S1- S4). The KMR diet consistently induced significant upregulation of genes central to ketogenesis and metabolic adaptation, demonstrating a robust shift toward ketogenic metabolism. In contrast, the CMR diet exhibited negligible or suppressive effects compared to the control diet (Table 7; Fig. 7).

Key enzymes of ketone body metabolism showed the most pronounced changes under KMR feeding. *Hmgcs2*, the rate-limiting enzyme in ketogenesis, displayed the highest fold-change with a 2.69-fold increase (*p < 0.001*), while *Bdh1*, essential for ketone body utilization, was elevated 1.64-fold (*p < 0.05*).In contrast, the CMR diet downregulated *Bdh1* (0.66-fold) and showed only a minor, non-significant upregulation of *Hmgcs2* (1.09-fold). These findings confirm that the KMR diet effectively activates hepatic ketone body synthesis and utilization, consistent with previous reports that ketogenic diets activate hepatic ketogenesis as a compensatory response to carbohydrate restriction^7,15^. Such transcriptional shifts are clinically relevant, given the therapeutic potential of ketogenic diets in metabolic disorders, obesity, type 2 diabetes, and neurological disorders^57,58^. The present findings extend these observations by showing that a ‎Moringa-supplemented KD not only induces ketogenesis but also sustains ketone utilization, a critical ‎factor for long-term metabolic health^59^. Notably, the CMR diet suppressed or blunted these pathways, suggesting that high-carbohydrate formulations may reduce metabolic adaptability, as supported by Falamarzi, et al. ^60^.

Metabolic regulatory genes (*Sirt3* and *Fgf21*) were also strongly affected. Sirt3 expression increased 2.39-fold (*p < 0.01*) under KMR feeding, consistent with improved mitochondrial function and oxidative stress regulation. *Fgf21*, a hepatokine critical for systemic metabolic adaptation, rose 1.65-fold (*p < 0.05*), supporting enhanced lipid mobilization and energy regulation. The CMR group, however, showed no significant effect on *Sirt3* (1.35-fold, ns) and significantly suppressed *Fgf21* expression (0.65-fold, *p* < 0.05).

The strong induction of *Sirt3* under KMR feeding suggests improved mitochondrial efficiency and oxidative stress regulation, in line with findings that ketogenic diets enhance mitochondrial metabolism through *Sirt3*-mediated deacetylation^61^. Similarly, *Fgf21* upregulation reinforces its established role as a master regulator of lipid mobilization and energy balance during carbohydrate restriction^62^. Importantly, the CMR diet downregulated *Fgf21*, indicating impaired systemic metabolic signaling compared to KMR. These findings suggest that *Moringa*’s bioactive compounds (e.g., flavonoids) may synergize with the KD to amplify *Sirt3/Fgf21* pathways, though this requires further validation.

A Novel and unexpected upregulation of *IL10* in the KMR group was noted (1.76-fold, *p* < 0.05). While ketogenic diets are known for anti-inflammatory properties via NLRP3 inflammasome inhibition^63^, our data suggest an additional mechanism mediated by *Moringa oleifera* phytochemicals, such as flavonoids and quercetin. This aligns with Fard, et al. ^64^, who reported *IL10* induction by *M. oleifera* extracts. Thus, the immunomodulatory effect observed here may represent the benefit of the ketogenic framework, a finding that has not been reported previously.

The *Moringa oleifera*–supplemented KMR induces strong transcriptional adaptations that enhance ketogenesis, mitochondrial function, metabolic flexibility, and anti-inflammatory signaling, largely driven by the combined effects of ketosis and moringa. Unlike the CMR, which exhibits minimal or suppressive effects, KMR combines the metabolic benefits of ketosis with phytochemical-mediated immune modulation, particularly through the upregulation of IL-10. This dual mechanism positions KMR as a promising dietary strategy for obesity, type 2 diabetes, and neuroinflammatory disorders, warranting further validation at the protein and functional levels. Moreover, a limitation of this study is the absence of glucagon measurements. Future investigations should include comprehensive hormonal profiling (glucagon, cortisol, growth hormone, and other counter-regulatory hormones) to define better the endocrine mechanisms underlying the observed *metabolic responses.”*

**Table 7:** Relative gene expression and fold change of key regulatory nodes in ketogenic metabolism and inflammatory responses in liver tissue from female C57BL/6 mice fed different diets.

Diet	Bdh1		Hmgcs2		Sirt3		Fgf21		IL10	
	ΔΔCt	Fold Change	ΔΔCt	Fold Change	ΔΔCt	Fold Change	ΔΔCt	Fold Change	ΔΔCt	Fold Change
CD	0.00	1	0	1.00	0.00	1.00	0.00	1.00	0.00	1.00
CMR	0.60	0.66 (↓)	-0.12	1.09 (↑, ns)	-0.43	1.35 (↑, ns)	0.63	0.65(↓)	0.31	0.80 (↓, ns)
KMR	-0.71	1.64 (↑*)	-1.43	2.69 (↑*)	-1.26	2.39 (↑**)	-0.72	1.65(↑*)	-0.81	1.76 (↑*)

*Bdh1*: 3-hydroxybutyrate dehydrogenase, type 1, *Fgf21*: Fibroblast growth factor 21, *Hmgcs2*: 3-hydroxy-3-methylglutaryl-Coenzyme A synthase 2, *Sirt3*: Sirtuin 3, *Il10*: Interleukin 10, *Actb*: Beta actin was used as a reference gene. Mice were fed on a basal diet (Control), Commercial Meal Replacement (CMR), and Ketogenic Meal Replacement (KMR). *ns = non-significant; ↑ = upregulation; ↓ = downregulation; *p < 0.05, **p < 0.01.

## Conclusion

A *Moringa oleifera*–enriched ketogenic meal replacement (KMR) demonstrates broad systemic benefits compared to a commercial meal replacement (CMR). Biochemically, KMR group showed the lowest body weight gain, improved lipid profile, and preserved insulin sensitivity. Although body weights remained stable across groups, the favorable lipid remodeling in KMR (e.g., elevated HDL and reduced LDL) underscores its potential to improve metabolic health independently of weight loss, aligning with ketogenic diet mechanisms. Histological examinations of all studied groups showed some noticeable changes in liver and kidney tissues. At the genetic level, KMR upregulated key ketogenic (*Bdh1*,* Hmgcs2*), mitochondrial regulatory genes (*Sirt3*,* Fgf21*), and the anti-inflammatory cytokine IL-10, supporting both metabolic reprogramming and immune modulation. Together, these adaptations promote ketogenesis, mitochondrial homeostasis, and systemic metabolic flexibility. Collectively, these findings position Moringa-supplemented KMR as a superior dietary strategy with potential application in obesity and type 2 diabetes, warranting long-term and clinical evaluation.

## Supplementary Information

Below is the link to the electronic supplementary material.

Supplementary Material 1

Supplementary Material 2

Supplementary Material 3

Supplementary Material 4

## Data Availability

The authors declare that the data supporting the findings of this study are available within the paper and its Supplementary Information files (S1- S4). Should any raw data files be needed in another format, they are available from the corresponding author upon reasonable request.
