# Supplementary material for: Metabolic and molecular evaluation of Moringa oleifera-supplemented ketogenic meal replacement in healthy C57BL/6 mice
Source: Sci Rep. 2026 Jan 28;16:4091. doi: 10.1038/s41598-025-34443-z (PMC12855186; doi:10.1038/s41598-025-34443-z)
Supplement: Supplementary file 1 — Supplementary Material 1 [file 41598_2025_34443_MOESM1_ESM.pdf]

truncated example of Matrix with non-normalized data (raw Ct values for target gene)

ID_REF	SAMPLE 1 (CD)	SAMPLE 2 (CMR)	SAMPLE 3 (KMR)
Bdh1_Ct	30.34	31.81	31.02
Bdh1_Ct	30.32	31.8	30.96
Bdh1_Ct	30.03	31.52	30.93
Bdh1_Ct	30.05	31.56	30.89
Bdh1_Ct	30.07	31.59	31.25
Bdh1_Ct	30.19	31.71	31.22
Bdh1_Ct	30.18	31.7	31.1
Bdh1_Ct	30.19	31.71	31.08
Hmgcs2_Ct	29.88	30.69	30.96
Hmgcs2_Ct	29.95	30.66	30.97
Hmgcs2_Ct	29.88	30.68	30.94
Hmgcs2_Ct	29.93	30.63	30.43
Hmgcs2_Ct	29.89	30.61	30.42
Hmgcs2_Ct	29.9	30.67	30.41
Hmgcs2_Ct	29.9	30.66	30.69
Hmgcs2_Ct	29.84	30.67	30.66
Sirt3_Ct	32.73	32.32	32.75
Sirt3_Ct	30.71	32.31	32.73
Sirt3_Ct	30.82	32.26	32.69
Sirt3_Ct	30.79	32.51	32.97
Sirt3_Ct	30.7	32.45	32.92
Sirt3_Ct	31.77	32.41	32.96
Sirt3_Ct	31.7	32.44	32.86
Sirt3_Ct	30.8	32.47	32.84
Fgf21_Ct	28.68	30.49	30.75
Fgf21_Ct	28.65	30.51	30.69
Fgf21_Ct	28.67	30.52	30.72
Fgf21_Ct	29.18	30.51	30.33
Fgf21_Ct	29.1	30.51	31.07
Fgf21_Ct	29.19	30.5	30.54
Fgf21_Ct	28.93	30.43	30.52
Fgf21_Ct	28.9	30.49	30.55
IL10_Ct	31.68	33.68	33.45
IL10_Ct	31.65	33.65	33.5
IL10_Ct	31.62	33.63	33.47
IL10_Ct	32.24	32.41	33.46
IL10_Ct	32.19	32.38	33.14
IL10_Ct	32.21	32.36	33.16
IL10_Ct	31.96	33.05	33.32
IL10_Ct	31.92	33.03	33.33
Actb_Ct	25.93	26.57	27.64
Actb_Ct	25.92	26.55	27.66
Actb_Ct	25.94	26.51	28.35
Actb_Ct	25.91	26.5	27.59
Actb_Ct	25.92	26.49	27.99
Actb_Ct	25.93	25.54	27.94
Actb_Ct	25.94	25.52	28.31
Actb_Ct	26.58	25.55	28.29
