# Supplementary material for: Metabolic and molecular evaluation of Moringa oleifera-supplemented ketogenic meal replacement in healthy C57BL/6 mice
Source: Sci Rep. 2026 Jan 28;16:4091. doi: 10.1038/s41598-025-34443-z (PMC12855186; doi:10.1038/s41598-025-34443-z)
Supplement: Supplementary file 2 — Supplementary Material 2 [file 41598_2025_34443_MOESM2_ESM.pdf]

truncated example of Matrix with PCR assay annotation columns and normalized target gene signal data

ID_REF	Unigene	GB_ACC	Symbol	Descsription	Gene Name	SPOT_ID	SAMPLE 1	SAMPLE 2	SAMPLE 3
Bdh1_Ct	71911	NM_00112268 3.1	Bdh1	3-Hydroxybutyrate dehydrogenase, type 1	Bdh1		0	0.6	-0.71
Hmgcs2_Ct	15360	NM_008256	Hmgcs2	3-Hydroxy-3-methylglutaryl-CoA synthase 2	Hmgcs2		0	-0.12	-1.43
Sirt3_Ct	64384	NM_00117780 4	Sirt3	Sirtuin 3, mitochondrial	Sirt3		0	-0.43	-1.26
Fgf21_Ct	56636	NM_020013	Fgf21	Fibroblast growth factor 21	Fgf21		0	0.63	-0.72
IL10_Ct	16153	NM_010548	Il10	Interleukin 10	IL10		0	0.31	-0.81
