# Supplementary material for: Metabolic and molecular evaluation of Moringa oleifera-supplemented ketogenic meal replacement in healthy C57BL/6 mice
Source: Sci Rep. 2026 Jan 28;16:4091. doi: 10.1038/s41598-025-34443-z (PMC12855186; doi:10.1038/s41598-025-34443-z)
Supplement: Supplementary file 3 — Supplementary Material 3 [file 41598_2025_34443_MOESM3_ESM.pdf]

SERIES

**title**	Evaluation of a Moringa oleifera-Supplemented Ketogenic Meal Replacement Diet in Healthy C57BL/6 Mice: A Real-time Quantitative PC
**summary**	This study evaluated the physiological, biochemical, histological, and genetic impacts of a Moringa oleifera–supplemented ketogenic meal replacement (KMR) compared with a commercial non-ketogenic meal replaceme
**summary**	Results showed that KMR-fed mice exhibited approximately 30% lower weight gain than both control and CMR groups. At the molecular level, KMR upregulated ketogenesis genes (Hmgcs2, Bdh1), mitochondrial regulato
**overall design**	qPCR gene expression profiling. Three experimental groups of female C57BL/6J mice (n=8/group): Control (standard chow), CMR (commercial meal replacement), and KMR (ketogenic meal replacement with Moringa ol
**contributor**	Hesham Elhariry, Department of Food Science, Faculty of Agriculture, Ain Shams Universi
**contributor**	Naglaa M. Ebeed, Department of Genetics, Faculty of Agriculture, Ain Shams University
**contributor**	Amr Fatouh, Department of Food Science, Faculty of Agriculture, Ain Shams University
**contributor**	Ahmed Ibrahim Hassaan, Department of Food Science, Faculty of Agriculture, Ain Shams

SAMPLES

# The Sample names in the first column are arbitrary but they must match the column headers of the Matrix table (see next worksheet).

Sample name	title	description	source name	organism	characteristics: treatment	characteristics: tissue	characteristics: strain	characteristics: sex	characteristics: age	molecule	label	platform
SAMPLE 1	Control	Liver tissue from fer	Female C57BL/6J m	Mus musculus	standard chow di	liver	C57BL/6J	female	6-8 weeks	total RNA	SYBR Gr	Custom_RTqPCR
SAMPLE 2	CMR	Liver tissue from fer	Female C57BL/6J m	Mus musculus	commercial meal	liver	C57BL/6J	female	6-8 weeks	total RNA	SYBR Gr	Custom_RTqPCR
SAMPLE 3	KMR	Liver tissue from fer	Female C57BL/6J m	Mus musculus	ketogenic meal re	liver	C57BL/6J	female	6-8 weeks	total RNA	SYBR Gr	Custom_RTqPCR

PROTOCOLS

**growth protocol**	Female C57BL/6 mice (6-8 weeks old; 20–22 g) obtained from National Research Centre (NRC), Cairo, Egypt. Housed in standard polypropylene cages under controlled conditions (25 ± 2°C, 55 ± 5% humidity, 12-h light
**treatment protocol**	After acclimatization, mice randomly divided into three groups (n=8/group) and fed different diets for 20 weeks ad libitum: Control (standard chow diet), CMR (commercial meal replacement), KMR (ketogenic meal replace
**extract protocol**	After 20-week treatment, mice fasted overnight then anesthetized with ketamine (120 mg/kg) and xylazine (16 mg/kg). Liver tissue excised, snap-frozen in liquid nitrogen, and stored at -80°C. Total RNA isolated using RN
**label protocol**	Reverse transcription performed using miScript II RT Kit (Qiagen) following manufacturer's instructions. cDNA synthesized from 1 µg total RNA and stored at -20°C until RT-qPCR analysis.
**hyb protocol**	n/a
**scan protocol**	n/a
**data processing**	RT-qPCR performed using Rotor Gene SYBR Green PCR Kit (Qiagen, USA). PCR conditions: initial denaturation at 95°C for 10 min, followed by 45 cycles of denaturation at 95°C for 20s, annealing at 59°C for 20s, and e
**value definition**	Raw Ct values obtained from RT-qPCR. Normalized expression calculated using 2^(-ΔΔCt) method where ΔCt = target gene Ct - reference gene (Actb) Ct, and ΔΔCt = ΔCt sample - mean ΔCt control group. Fold change

PLATFORM

If your panel of PCR assays **is** already deposited in GEO, please fill in **platform** column in **SAMPLES** section and ignore this **PLATFORM** section.
If your panel of PCR assays **is not** in GEO, please complete the fields below and include platform annotation columns in **Matrix normalized Template** (see example).

**title**	Custom RT-qPCR Platform for Ketogenesis and Metabolic Gene Analysis in Mouse Liver
**technology**	RT-PCR
**distribution**	virtual
**organism**	Mus musculus
**manufacturer**	Custom design using Qiagen reagents
**manufacture protocol**	see manufacturer's website
**catalog number**	Qiagen Cat no. / ID. 74134
**description**	Custom RT-qPCR assay panel targeting genes involved in ketogenesis (Bdh1, Hmgcs2), mitochondrial function (Sirt3, Fgf21), and inflammation (Il10)
**description**	All primers designed for mouse genome with annealing temperature of 59°C and amplicon sizes ranging from 126-231 bp

# Platform Column Definitions: describe the contents of each platform column in the Matrix normalized Template

Gene_Symbol	Official NCBI gene symbol
Gene_Name	Full gene name and function
RefSeq_ID	NCBI RefSeq accession number
Forward_Primer	Forward primer sequence (5' to 3')
Reverse_Primer	Reverse primer sequence (5' to 3')

Amplicon_Size	Expected PCR product size in base pairs
Annealing_Temp	Optimized annealing temperature in Celsius

Gene_Symbol	Gene_N	Forward_Primer_5to3	Reverse_Primer_5to3	RefSeq_ID	Amplicon_bp	Tm_C	Function
Bdh1	3-hydro:	GTTAACAACGCAG	AACTTGGTGATGCA	NM_001122683.1	215	59	Ketone body utilization
Hmgcs2	3-hydro	GCTGCCAACTGGA	GTCGTACGCGTTCT	NM_008256	195	59	Ketogenesis rate-limiting enzyme
Sirt3	Sirtuin :	CCGACATTGTGTTC	TCAAGCTGGCAAA	NM_001177804	126	59	Mitochondrial deacetylase
Fgf21	Fibrobla	GGTACCTCTACAC	AAGTGAGGCGATC	NM_020013	208	59	Metabolic hormone
Il10	Interleu	CAGGGCCCTTTGC	CGGCTGGGGGATG	NM_010548	168	59	Anti-inflammatory cytokine
Actb	Beta act	AGCCATGTACGTA	TCCCTCTCAGCTG	NM_007393	231	59	Reference gene
