# Supplementary material for: Metabolic and molecular evaluation of Moringa oleifera-supplemented ketogenic meal replacement in healthy C57BL/6 mice
Source: Sci Rep. 2026 Jan 28;16:4091. doi: 10.1038/s41598-025-34443-z (PMC12855186; doi:10.1038/s41598-025-34443-z)
Supplement: Supplementary file 4 — Supplementary Material 4 [file 41598_2025_34443_MOESM4_ESM.pdf]

truncated example of Matrix with fold change data (test/control)

ID_REF	SAMPLE 2/SAMPLE 1	SAMPLE 3/SAMPLE 1
Bdh1	0.66	1.64
Hmgcs2	1.09	2.69
Sirt3	1.35	2.39
Fgf21	0.65	1.65
IL10_Ct	0.8	1.76
